# A cross sectional evaluation of an alcohol intervention targeting young university students

**DOI:** 10.1186/s12889-016-3314-4

**Published:** 2016-07-20

**Authors:** Sharyn Burns, Jonine Jancey, Gemma Crawford, Jonathan Hallett, Linda Portsmouth, Janelle Longo

**Affiliations:** School of Public Health, Curtin University, GPO Box U1987, Perth, WA 6845 Australia; South Metropolitan Population Health Unit, Department of Health, PO Box 546, Fremantle, WA 6959 Australia

**Keywords:** Alcohol, University students, AUDIT, Alcohol-related harms, Alcohol-related problems, Alcohol expectancies

## Abstract

**Background:**

Hazardous drinking has been found to be higher among young university students compared to their non-university peers. Although young university students are exposed to new and exciting experiences, including greater availability and emphasis on social functions involving alcohol there are few multi strategy comprehensive interventions aimed at reducing alcohol-related harms.

**Methods:**

Random cross sectional online surveys were administered to 18–24 year old students studying at the main campus of a large metropolitan university in Perth, Western Australia. Prior to the completion of the second survey an alcohol intervention was implemented on campus. Completed surveys were received from 2465 (Baseline; T1) and 2422 (Post Year 1: T2) students. Students who consumed alcohol in the past 12 months were categorised as low risk or hazardous drinkers using the Alcohol Use Disorders Identification Test (AUDIT). Due to the cross sectional nature of the two samples two-tailed two-proportion *z*-test and two sample *t*-tests were employed to determine statistical significance between the two time periods for categorical and continuous variables respectively.

**Results:**

At T1 and T2 89.1 % and 87.2 % of the total sample reported drinking alcohol in the past month respectively. Hazardous levels of alcohol consumption reduced slightly between T1 (39.7 %) and T2 (38 %). In both time periods hazardous drinkers reported significantly higher mean scores for experienced harm, second-hand harm and witnessed harm scores compared to low risk drinkers (*p* <0.001). Hazardous drinkers were significantly more likely to experience academic problems due to their alcohol consumption and to report more positive alcohol expectations than low risk drinkers at both time periods (*p* <0.001).

**Conclusions:**

Harms and problems for students who report hazardous drinking are of concern and efforts should be made to ensure integrated and targeted strategies reach higher risk students and focus on specific issues such as driving while intoxicated and alcohol related unplanned sexual activity. However there is also a need for universal strategies targeting all students and low risk drinkers as they too are exposed to alcohol harms within the drinking and social environment. Changing the culture of the university environment is a long term aim and to effect change a sustained combination of organisational actions, partnerships and educational actions is required.

## Background

The transition period from secondary school to college or university has been identified as a particularly high risk period for a range of health compromising behaviours, including excessive alcohol consumption [1, 2]. Many young university students drink alcohol at levels that place themselves and others at risk of a range of short and long term harms [3–7]. Hazardous drinking has been found to be higher among young university students in New Zealand compared to their non-university peers [8] with suggestions that the university environment contributes to these differences [8, 9]. Young university students are exposed to new and exciting experiences, including greater availability and emphasis on social functions involving alcohol [8]. This is an important developmental period during which many young people explore their identity and form more mature relationships [1]. In addition to these changes some students live away from their family home for the first time [8].

Significant increases in proportions of 12–17 year olds in Australia choosing to abstain from alcohol and fewer young people exceeding adult guidelines for single occasion risk between 2010 and 2013 demonstrate encouraging changes in alcohol consumption for school-aged students. However despite these encouraging findings young people aged 18–24 years were more likely to drink at harmful levels on a single occasion than other adult age groups [10]. These data support the ongoing need to provide positive and effective strategies to reduce levels of alcohol consumption and associated harm as young people move to tertiary education and the workforce.

Although the university has been identified as an ideal setting for health promotion interventions [2, 11], there is paucity of integrated, comprehensive interventions focusing on reducing alcohol-related harms among Australian universities described in the literature. Despite this, there have been interventions focusing on a single strategy, usually brief interventions, that have demonstrated some short term changes in alcohol consumption levels [12, 13] and alcohol related problems [12] however no significant differences were reported for alcohol-related harms [13].

This paper describes a university based alcohol intervention and compares low risk and hazardous drinking prevalence and experienced, second-hand, witnessed and academic harms for the total sample, and makes comparisons between low risk and hazardous drinkers at baseline and after the first year of the intervention. The paper will describe the effect of the intervention after year one.

### Theoretical basis of the youth alcohol project intervention

The Youth Alcohol Project (YAP) was implemented at a large and culturally diverse university campus in Australia with the aim of reducing the witnessed and experienced harms associated with alcohol consumption among 18–24 year old students. Social Cognitive Theory (SCT) was used to inform the development of the intervention. The underlying premise of SCT is reciprocal determinism, in particular the relationship between the individual, behaviours and the environment [14]. Individual traits such as personality, genetic factors and gender have been found to influence alcohol consumption and related behaviours [15–17]. The environment is a significant influence in initiation and drinking behaviours of young people [16, 17]. The university environment which may include events that encourage excessive drinking and new peer networks [8] along with the belief that excessive alcohol use is a ‘rite of passage’ [18] and an integral part of the university experience [19] are important influences. Social and cultural norms which suggest for some young people drinking to get drunk is the main goal of many events and social occasions [20, 21] are often influenced by alcohol expectancies which are formed through social influences including family, peers and culture [22]. SCT recognises the power of observational learning such as the actions of peers and significant others [14] which is supported in the alcohol literature, recognising drinking behaviours are strongly influenced by the behaviours of peers, siblings and other family [15]. Norms and expectations help reinforce physical aspects of the environment including availability, promotion of alcohol and poorly implemented policy which together encourage excessive alcohol consumption [8, 15].

#### The intervention

**Fig. 1:**
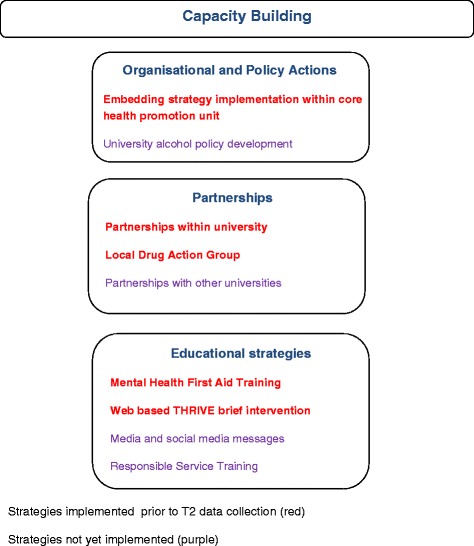
Intervention Strategies

The YAP was implemented using a multi-strategy staged approach. At year one (T1) data analysis some strategies had been only partially implemented. Commitment to a capacity building approach was adopted to embed strategies into university structures. Capacity building involves processes that build infrastructure, program sustainability and work to skill and empower individuals and groups [23, 24]. The intervention includes a focus on organisational actions, partnerships and education actions. The strategies of the intervention are identified in Fig. 1 with their implementation status highlighted.

To coordinate and provide effective support to strategy implementation [24], partnerships were established with the Guild (student body), student support services, health services, security and housing personnel, campus venues such as taverns, cafes and sports clubs with licences to serve alcohol. These partnerships have worked to ensure more responsible promotion of alcohol on campus. The initiation and maintenance of a Local Drug Action Group (LDAG) (see http://localdrugaction.com.au) provides on going opportunities for community action, advocacy and education. During the first year of the intervention the LGAG produced an educational wallet card with first aid strategies for helping intoxicated friends and supported the Mental Health First Aid (MHFA) strategy.

Specific training, such as Responsible Service of Alcohol (RSA) training and MHFA were implemented to enhance the skills of the student community [25]. Provision of face-to-face *Responsible Service of Alcohol* training courses enhances skills and employability of students. These courses provide an additional opportunity for advocacy for responsible service, especially among clubs and groups. The adoption of responsible service practices was found to reduce levels of high risk drinking at community sporting clubs [26]. During the first year of the intervention two face-to-face RSA trainings were conducted with university sports club committee members (*n* = 30). Young university students have been found to experience higher levels of mental health problems than their peers [27] and high comorbidity with mental health problems and harmful alcohol consumption [28] reinforcing the importance of integrated strategies. MHFA aims to improve mental health literacy and to develop skills and confidence to provide help and referral in a mental health crisis or for ongoing mental health problems, including those related to alcohol and other drug use. The program has previously demonstrated effectiveness in the community [29], among university students [30] and workplaces [31]. During the first year of the intervention 295 students received MHFA training.

The web-based THRIVE (Tertiary Health Research Intervention Via Email) alcohol brief intervention, developed through this university and evaluated through a randomised controlled trial [13, 32], was updated and integrated into the university website to provide an easily accessible and anonymous intervention for students. The program is a brief motivational health intervention consisting of an online alcohol assessment that delivers immediate and personalised feedback to participants on drinking behaviour, risks of harm, strategies for reducing consumption, and available support services for those drinking at harmful levels [13]. THRIVE is embedded in the student web portal and is voluntarily accessed. The program’s referral system supports student transition to other existing interventions and alcohol and other drug counselling services provided through campus health services. Promotional strategies including the development of a bookmark distributed at campus events and web-based promotion were implemented to enhance the awareness of THRIVE.

To improve skills and empower young people the development of intervention strategies have been embedded into a core health promotion unit for undergraduate students. Students worked in small groups to develop, implement and evaluate a number of organisational actions, partnerships and educational actions. Embedding strategies within a unit supports sustainability and a committed group of project officers which stretches limited health promotion dollars further [33]. In the first year students worked on a range of projects linked to the ongoing strategies of the YAP.

The YAP is currently planning educational strategies with a focus on media and social media. Six focus groups were conducted with key stakeholders from the Tavern (*n* = 5), Security (*n* = 6) and students (four groups; *n* = 35 students) to inform the development of educational strategies. Educational actions can be effective in improving knowledge, attitudes, skills and behaviours [23]. Based on the findings of the focus groups the intervention will utilise social media to facilitate positive change during the second year of the intervention Policy interventions are often integral to positive changes in health behaviour [34, 35]. The development and implementation of an alcohol policy is a current focus of the intervention.

## Methods

Random cross sectional online surveys were conducted during July-August 2013 (baseline: T1) and 2014 (year 1: T2) in one university. The YAP commenced implementation after T1 data collection. For both time periods 6000 students aged 18–24 years were emailed via their student email address by the University Surveys Office to invite them to participate in the study. Inclusion criteria required that the respondents’ be studying at the main campus of the university and enrolled internally. In addition, random intercept surveys, administered by trained research assistants and completed online via i-pad, were conducted on campus market day (food and market stalls and activities) during the data collection period. Due to costs associated with data collection both surveys were cross sectional and a specific cohort was not followed. This study was approved by the Curtin University Human Ethics Committee (Approval no. HR 54/2013).

### Instrumentation

Students were asked if they had drunk alcohol during the last 12 months [36]. Students who responded ‘no’ to this question did not complete the 10 item Alcohol Use Disorders Identification Test (AUDIT) [37] and the Alcohol Problems Scale [38] questions.

Consistent with other Australian studies the AUDIT scores were computed to binary variables low risk (non-hazardous; < 8) and hazardous (risky; ≥ 8) drinking [5, 6, 13] to measure level of alcohol-related harm and consumption.

Harms experienced in the past 12 months were measured by the Alcohol Problems Scale, a 17 item scale of harms as a result of personal alcohol consumption) [4, 38]. Students responded yes, no or prefer not to answer [score range 0–17] (See Table 4 for specific items). Second-hand harm, harms as a result of other students drinking during the past 4 weeks were measured using an 11 item scale [38, 39]. Witnessed harms, harms witnessed as a result of other students drinking during the past 4 weeks included a scale comprised of six harms [40]. For second-hand and witnessed harms students were provided with responses ranging from never to four or more times [score range 0–44 and 0–24 respectively with 0 representing no harm] (see Table 5 for specific items for second-hand and witnessed harms) [40].

The Academic Role Expectations and Alcohol Scale (AREAS) [38] included four items with responses ranging from not at all to four or more times during a four week reference period [score range 0–16]. The Brief Comprehensive Effects of Alcohol Scale (B-CEOA) [41] was used to measure alcohol expectancies. The scale included nine items with responses agree, neither agree or disagree or disagree [score range 9–27] (see Table 6 for specific items for AREAS and B-CEOA). Proportion of friends who regularly drink alcohol was measured to determine the influence of close peers on alcohol consumption.

Demographic data included age, gender, international or domestic student status, Faculty of enrolment (Business, Engineering and Science, Health Science or Humanities or Centre for Aboriginal Studies) and place of residence while at university (living in a shared house, with a parent or guardian, as a boarder or alone or with partner/children). The questionnaire was tested for face validity (*n* = 10) and content validity (*n* = 8). Test-retest was conducted with a purposive sample of the target group (*n* = 60). A detailed discussion of the development of this questionnaire and description of the variables can be found elsewhere [40].

### Data analysis

The dependent variable for this study was the binary AUDIT score of low risk and hazardous drinking. Chi square analysis was used to determine statistical significance and proportions for categorical variables to compare low risk and hazardous drinkers at data collection periods. Analysis of Variance (ANOVA) was used to compute means and to determine statistical significance for continuous variables at each time period. Continuous variables included experienced, second-hand and witness harms, academic problems and alcohol expectancies. A two-tailed two-proportion z-test and two sample *t*-tests were employed to determine statistical significance between the two time periods for categorical and continuous variables respectively. Highly and moderately significant differences were discerned by *p*-values of *p* < 0.001 and *p* < 0.05 respectively [42].

## Results

### Demographics

At T1 1930 students responded to the online survey (32.2 % response rate) and a further 628 were recruited via intercept. At T2, 1825 (30.4 % response rate) responded online and 681 via intercept modes respectively. Completed surveys were received from 2465 (T1) and 2422 (T2) students. There was no significant difference between data collected online or via random intercept at T1 or T2.

Between T1 and T2 there was no significant difference between the proportions of students who responded in regard to gender, age, international or domestic student status, Faculty or place of residence (apart from slightly more students living with parents at T2 (*p* <0.05). For both data collection time periods females were more likely to respond (62.1 %) (Table 1).

**Table 1:** Demographics for the total sample at baseline and post 1

	Baseline (T1) (2013)	Post 1 (T2) (2014)	significance (p) T1/T2
Gender			
Male	926 (37.6)	908 (37.5)	0.968
Female	1531 (62.1)	1504 (62.1)	0.992
Other	8 (0.3)	9 (0.4)	0.779
Total	2465	2421	
Age			
18–20 years	1191 (48.3)	1208 (49.9)	0.271
21–24 years	1275 (51.7)	1214 (50.1)	0.200
Total	2466	2422	
International/domestic student status			
International student	300 (12.2)	283 (11.6)	0.603
Domestic student	2166 (87.8)	2139 (88.3)	0.603
Total	2466	2422	
Faculty			
Health Science	918 (37.5)	876 (36.2)	0.441
Science and Engineering	552 (22.4)	541 (22.3)	0.968
Humanities	494 (19.6)	537 (22.2)	0.067
Business	496 (19.7)	462 (19.1)	0.363
Aboriginal Studies	6 (0.2)	6 (0.2)	0.976
Total	2466	2422	
Place of residence while at university			
Share flat/house	559 (23.5)	590 (25.1)	0.190
Student housing	105 (4.4)	114 (4.9)	0.465
Parent/guardian	1507 (63.3)	1418 (60.3)	0.038**
Live alone	39 (1.6)	46 (2)	0.406
With partner/children	114 (4.8)	128 (5.4)	0.303
Board/live with other relative or friend/other	58 (2.5)	54 (2.3)	0.756
Total	2382	2350	

*(*p* <0.001)

**(*p* <0.05)

### Hazardous vs low risk drinkers

There were some significant differences in some demographics when current drinkers were categorised as low risk drinkers (< 8 AUDIT score) or hazardous drinkers (> 8 AUDIT score) (Table 2). At T2 older students (21–24 years) were less likely to be categorised as hazardous drinkers and more likely to be low risk drinkers compared to T1. There were significantly less international students and significantly more domestic students who reported hazardous drinking at T2 and significantly less domestic students who reported low risk drinking at T2.

**Table 2:** Demographics, harms and influencing factors for low risk and hazardous drinkers at the two time periods

	T1				T2			
	Low risk	Hazardous	Total	*P* value	Low risk	Hazardous	Total	*P* value
	*N* (%)	*N* (%)	*N* (%)		*N* (%)	*N* (%)	*N* (%)	
Gender				0.000				0.000**
Male	407 (53.1)	359 (46.9)	766		405 (57.5)	299 (42.5)	704 (37.3)	
Female	823 (64.8)	447 (35.2)	1270		761 (65)	413 (35)	1174 (62.2)	
Other	0	4 (100)	4		3 (37.5)	5 (62.5)	8 (0.4)	
Total	1230	810	2040		1169	717	1886	
Age				0.528				0.004*
18–20 years	606 (61)	388 (39)	994 (48.7)		563 (59)	391 (41)	954 (50.6)	
21–24 years	624 (59.6)	423 (40.4)	1047 (51.3)		607 (65)*	326 (35)*	933 (49.4)	
Total	1230	811	2041		1170	717	1887	
International Students				0.000				0.000**
International	160 (74.8)	54 (25.2)	214 (10.5)		157 (88.2)*	21 (11.8)*	178 (9.4)	
Domestic	1070 (58.6)	757 (41.4)	1827 (89.5)		1013 (59.3)	696 (40.7)	1709 (90.6)	
Total	1230	811	2041		1107	717	1887	
Faculty				0.285				0.116
Health Science	474 (61.5)	297 (38.5)	771 (37.2)		429 (62.5)	257 (37.5)	686 (36.4)	
Science and Engineering	262 (58)	190 (42)	452 (22.4)		245 (59.8)	165 (40.2)	410 (21.7)	
Humanities	246 (58.6)	174 (41.4)	420 (20)		285 (66)*	147 (34)	432 (22.9)	
Business	247 (62.7)	147 (37.3)	394 (20.1)		210 (59.2)	145 (40.8)	355 (18.8)	
Aboriginal Studies	1 (25)	3 (75)	4 (0.2)		1 (0.1)	3 (0.4)	4 (0.2)	
Total	1230	811	2041		1170	717	1887	
Place of residence while at university				0.279				0.000**
Share flat/house	275 (58)	199 (42)	474		274 (23.4)	209 (29.1)*	483 (25.6)	
Student housing	57 (63.3)	33 (36.7)	90		46 (3.9)	44 (6.1)	90 (4.8)	
Parent/guardian	786 (60.6)	510 (39.4)	1296		728 (62.2)	407 (56.8)*	1135 (60.1)	
Live alone	21 (68.4)	12 (34.6)	33		19 (1.6)	13 (1.8)	32 (1.7)	
With partner/children	67 (68.4)	31 (31.6)	98		82 (7.0)	25 (3.5)	107 (5.7)	
Board/live with other relative or friend/other	24 (48)	26 (52)	50		21 (1.8)	19 (2.6)	40 (2.2)	
Total	1230	811	2041		1170	717	1887	
Experienced harm	M2.453	M 5.6662	M 3.596	0.000	M 2.089	M6.0713-	M 3.5958	0.000**
	SD 2.453	SD 3.088	SD 3.20		SD 2.394	SD 3.2023	SD 3.3421	
	CI 2.079–2.357	CI 5.451–5.881	CI 4.455–3.736		CI 1.951–2.227	CI 5.833–6.308	CI 3.443–3.748	
Second-hand harm	M 1.305	M 3.221	M 2.070	0.000	M 1.284	M 3.906	M 2.279	0.000**
	SD 2.550	SD 4.660	SD 3.668		SD 2.525	SD 5.720	SD 4.240	
	CI 1.61–1.44	CI 2.89–3.54	CI 1.919–2.231		CI 1.134–1.425	CI 3.471–4.342	CI 2.080–2.478	
Witnessed harm	M 1.476	M 3.148	M 2.144	0.000	M 1.279	M 3.160	M 1.990	0.000**
	SD 2.841	SD 4.101	SD 3.497		SD 2.479	SD 4.036	SD 3.287	
	CI 1.316–1.636	CI 2.864–3.431	CI 1.992–2.296		CI 1.136–1.422	CI 2.861–3.459	CI 1.841–2.140	
Academic problems	M 0.4891	M 2.555	M 1.314	0.00	M 0.489	M 2.798	M 1.363	0.000**
	SD 1.454	SD 3.578	SD 2.721		SD 1.461	SD 3.726	SD 2.798	
	CI 0.406–0.571	CI 2.307–2.804	CI 1.195–1.434		CI 0.405–0.574	CI 2.522–3.075	CI 1.235–1.490	
Alcohol expectancies	M 21.110	M 23.312	M 21.985	0.000	M 20.817	M 23.555	M 21.857	0.000**
	SD 3.669	SD 2.920	SD 3.558		SD 3.990	SD 2.964	SD 3.869	
	CI 20.905–21.315	CI 23.110–23.513	CI 21.830–22.139		CI 20.588–21.0460	CI 23.337–23.772	CI 21.682–22.032	
Friends who drink regularly				0.000				0.000**
None	34 (2.9)	4 (0.5)	38 (1.9)		58 (5.1)**	4 (0.6)	62 (3.4)	
A few	432 (36.2)	87 (11)	519 (26.2)		428 (37.6)	65 (9.4)	493 (27.0)	
Half	217 (18.2)	76 (9.6)	293 (14.8)		192 (16.9)	87 (12.6)	279 (15.3)	
Most	431 (36.2)	431 (54.6)	862 (43.5)		394 (34.6)	358 (51.9)	752 (41.1)	
All	78 (6.5)	192 (24.3)	270 (13.6)		67 (5.9)	176 (25.5)	243 (13.3)	

*p <0.05 between T1 and T2; **p <0.001 between T1 and T2

### Reporting of alcohol consumption

At T1 89.1 % and at T2 87.2 % of the total sample reported drinking alcohol in the past month. Hazardous levels of alcohol consumption reduced slightly between T1 (39.7 %) and T2 (38 %) however these results were not statistically significant. There were no statistically significant differences in mean scores for a) experienced harms, b) witnessed and second-hand harms, c) academic problems or d) alcohol expectancies over the two time periods (Table 3). There were some moderately significant differences in the proportion of close friends who drank alcohol at each time period with more students reporting that none of their close friends drank alcohol (6.4 % T2 vs 4.5 % T1) and less students reporting most of their friends drank alcohol at T2 (40.7 % T1 vs 37.7 % T2) (*p* <0.05).

**Table 3:** Drinking levels, harms and friends alcohol consumption for total sample at T1 and T2

	Baseline (T1) (2013)	Post 1 (T2) (2014)	significance (p) T1/T2
Drunk alcohol in last 12 months			
Yes	2061 (89.1)	1905 (87.2)	0.051
No	252 (10.9)	279 (12.8)	0.051
Total	2313	2184	
AUDIT Score			
Low risk	1230 (60.3)	1170 (62)	0.262
Hazardous	811 (39.7)	717 (38)	0.262
Total	2041	1887	
Experienced harm score	M: 3.596	M: 3.618	0.963
Total *n*	1995	1853	
Second-hand harms score	M: 2.0495	M: 2.2270	0.999
Total *n*	2013	2013	
Witnessed harms score	M: 2.0500	M: 1.8968	0.970
Total *n*	2013	2136	
Academic problems score	M: 1.3034	M: 1.4544	0.963
Total *n*	1995	1853	
Alcohol Expectancies score	M: 21.5124	M: 21.2314	0.965
Total *n*	2041	2184	
Friends who drink regularly			
None	99 (4.5)	134 (6.4)	0.005**
A few	619 (27.9)	615 (29.3)	0.327
Half	322 (14.5)	308 (14.7)	0.896
Most	902 (40.7)	792 (37.7)	0.044**
All	275 (12.4)	252 (12)	0.681
Total	2217	2101	

*(*p* <0.001)

**(*p* <0.05)

### Low risk versus hazardous drinkers and associated harms

Further analyses were conducted using the dependent variable low risk and hazardous consumption at each time period (Table 3). When harms (experienced; second hand; witnessed) and academic problems were compared there were little differences in total mean scores for all current drinkers, at T1 compared to T2. In both time periods hazardous drinkers reported significantly higher mean scores for experienced harm, second-hand harm and witnessed harm scores compared to low risk drinkers (*p* <0.001). Of the total sample approximately 71 % had experienced hangovers at both time periods. Hazardous drinkers were significantly more likely than low risk drinkers to have experienced unprotected sex (Hazardous T1 34.5 %; T2 35.4 %; Low risk T1 9.35; T2 9.6 %)), had driven a car while intoxicated (Hazardous T1 28.7 %; T2 32 %; Low risk T1 9.2 %; T2 6.7 %), were a passenger in a car where the driver was intoxicated (Hazardous T1 39.6 %; T2 43.2 %; Low Risk 11.1 %; 10.7 %) and had been removed or banned from a club or pub because of their drinking (Hazardous T1 22.8 %; T2 23.1 %; Low Risk T1 5.4 %; T2 4 %) (Table 4). Hazardous drinkers were also significantly more likely than low risk drinkers to have experienced second-hand harms (as a result of other students drinking) such as being insulted or humiliated (Hazardous T1 28.5 %; T2 29.1 %; Low risk T1 12.5 %; T2 12.2 %) and taking care of another student who had drunk too much (Hazardous T1 44.4 %; T2 48.4 %; Low risk T1 27.5 %; T2 26.5 %)) (Table 5). Witnessing someone pass out (Hazardous T1 50.5 %; T2 26.4 %; Low risk T1 28.9 %; T2 26.4 %), a serious argument or quarrel (Hazardous T1 41 %; T2 21.3 %; Low risk T1 17.9 %; T2 15.7 %) and sexual assault (Hazardous T1 20 %; T2 9.7 %; Low risk T1 9.5 %; T2 9.7 %) was significantly more likely for hazardous drinkers compared to low risk drinkers at both time periods (Table 5).

**Table 4:** Experienced Harms and Level of Alcohol Consumption for Low Risk and Hazardous drinkers at T1 and T2

	T1 (*n* = 1995)			T2 (*n* = 1853)		
	Low risk	Hazardous	Total	Low risk	Hazardous	Total
	*N* (%)	*N* (%)	*N* (%)	*N* (%)	*N* (%)	*N* (%)
Hangover	692 (57.8)	729 (91.5)	1421 (71.2)^a^	668 (58)	647 (92.3)	1315 (71)^b^
Emotional outburst	321 (26.8)	457 (57.3)	778 (39)^a^	285 (24.7)	418 (59.6)	703 (37.9)^b^
Vomiting	486 (40.6)	584 (73.3)	1070 (63.6)	422 (36.6)	535 (76.3)	957 (51.6)^b^
Heated argument	139 (11.6)	315 (39.5)	454 (22.8)^a^	157 (13.6)	304 (43.4)	461 (24.9)^b^
Physically aggressive	45 (3.8)	162 (20.3)	207 (10.4)^a^	44 (3.8)	147 (21)	191 (10.3)^b^
Blackouts	286 (23.9)	567 (71.1)	853 (42.8)^a^	244 (21.2)	541 (77.2)	785 (42.4)^b^
Inability to pay bills	19 (1.6)	62 (7.8)	81 (4.1)^a^	14 (1.2)	67 (9.6)	81 (4.4)^b^
Unprotected sex	111 (9.3)	275 (34.5)	386 (19.3)^a^	111 (9.6)	248 (35.4)	359 (19.4) ^b^
Sexual situation unhappy about at time	57 (4.8)	118 (14.8)	175 (8.8)^a^	52 (4.5)	130 (18.5)	182 (9.8)^b^
Sexual encounter later regretted	98 (8.2)	237 (29.7)	335 (16.8)^a^	85 (7.4)	235 (33.5)	320 (17.3)^b^
Suffered an injury	20 (1.7)	74 (9.3)	94 (4.7)^a^	18 (1.6)	74 (10.6)	92 (5)^b^
Drove a car	110 (9.2)	229 (28.7)	339 (17)^a^	77 (6.7)	224 (32)	301 (16.2)^b^
Passenger in a car	133 (11.1)	316 (39.6)	449 (22.5)^a^	123 (10.7)	303 (43.2)	426 (23)^b^
Stole private or public property	39 (3.3)	110 (13.8)	149 (7.5)^a^	28 (2.4)	111 (15.8)	139 (7.5)^b^
Act of vandalism	21 (1.8)	81 (10.2)	102 (5.1)^a^	20 (1.7)	85 (12.1)	105 (5.7)^b^
Removed or banned from a pub or club	65 (5.4)	182 (22.8)	247 (12.4)^a^	46 (4)	162 (23.1)	208 (11.2)^b^
Arrested	16 (1.3)	18 (2.3)	34 (1.7)	13 (1.1)	25 (3.6)	38 (2.1)^b^

*T1* Significant between low risk and hazardous drinkers (*p* <0.001)^a^ *T2* Significant between low risk and hazardous drinkers (*p* <0.001)^b^

**Table 5:** Second-hand and Witnessed Harms and Level of Alcohol Consumption for Low Risk and Hazardous drinkers at T1 and T2

Second-hand Harms						
	T1 (*n* = 2103)			T2 (*n* = 1861)		
	Low risk	High risk	Total	Low risk	High risk	Total
	*n* (%)	*n* (%)	*n* (%)	*n* (%)	*n* (%)	*n* (%)
Been insulted or humiliated	149 (12.3)	229 (28.5)	378 (18.8)^a^	141 (12.2)	205 (29.1)	346 (18.6)^b^
Had a serious argument or quarrel	87 (7.2)	180 (22.4)	267 (13.3)^a^	93 (8)	187 (26.6)	280 (15)^b^
Been pushed, hit or otherwise assaulted	44 (3.6)	109 (13.6)	153 (7.6)^a^	35 (3)	105 (14.9)	140 (7.5)^b^
Had your property damaged	46 (3.8)	111 (13.8)	157 (7.8)^a^	52 (4.5)	119 (16.9)	171 (9.2)^b^
Had to baby-sit or take care of another student who had drunk too much	333 (27.5)	357 (44.4)	690 (34.3)^a^	307 (26.5)	341 (48.4)	648 (34.8)^b^
Found vomit in halls or bathroom of residence	83 (6.9)	117 (14.6)	200 (9.9)^a^	78 (6.7)	144 (20.5)	222 (11.9)^b^
Had studying or sleep interrupted	236 (19.5)	275 (34.2)	511 (25.4)^a^	187 (16.2)	258 (36.6)	445 (23.9)^b^
Experienced an unwanted sexual advance	103 (8.5)	178 (22.1)	281 (14)^a^	82 (7.1)	154 (21.9)	236 (12.7)^b^
Been a victim of sexual assault (including date rape)	11 (0.9)	25 (3.1)	36 (1.8)^a^	10 (0.9)	30 (4.3)	40 (2.1)^b^
Been a victim of another crime on campus	8 (0.7)	22 (2.7)	30 (1.5)^a^	11 (1)	30 (4.3)	41 (2.2)^b^
Been a victim of another crime off campus*	14 (1.2)	42 (5.2)	56 (2.8)^a^	77 (7.1)^a^	110 (16.4)	187 (10.6)^b^
Witnessed Harms						
	T1 (*n* = 2013)			T2 (*n* = 1861)		
	Low risk	High risk	Total	Low risk	High risk	Total
	*n* (%)	*n* (%)	*n* (%)	*n* (%)	*n* (%)	*n* (%)
Someone being pushed, hit or otherwise assaulted	217 (17.9)	271 (33.7)	488 (24.2)^a^	182 (15.7)	224(34.7)	426 (22.9)^b^
Serious argument or quarrel	274 (22.7)	330 (41)	604 (30)^a^	246 (21.3)	288 (40.9)	534 (28.7)^b^
Property damage	136 (11.2)	225 (28)	361 (17.9)^a^	150 (13)	194 (27.6)	344 (18.5)^b^
Someone pass out	349 (28.9)	406 (50.5)	755 (37.5)^a^	306 (26.4)	381 (54.1)	687 (36.9)^b^
Someone you suspect had alcohol poisoning	122 (10.1)	189 (23.5)	311 (15.4)^a^	109 (9.4)	148 (21)	257 (13.8)^b^
A sexual assault	115 (9.5)	161 (20)	276 (13.7)^a^	112 (9.7)	147 (20.9)	259 (13.9)^b^

*T1* Significant between low risk and hazardous drinkers (*p* <0.001)^a^ *T2* Significant between low risk and hazardous drinkers (*p* <0.001)^b^

Similarly hazardous drinkers were significantly more likely to experience academic problems at both time periods compared to low risk drinkers (*P* <0.001). For example, at T1 and T2 42.8 and 45.6 % of hazardous drinkers respectively indicated they had been unable to concentrate in class because of their drinking compared to 12.5 % (T1) and 12.7 % (T2) of low risk drinkers. Hazardous drinkers were more likely to report positive alcohol expectancies at both time periods (*p* <0.001). These students anticipated alcohol would enable them to act more sociably (Hazardous T1 86.4 %; T2 66.8 %; Low risk T1 72.4 %; T2 66.8 %) and to have fun/ a good time (Hazardous T1 81 %; T2 57.5 %; Low risk T1 61.5; T2 57.5 %) (Table 6).

**Table 6:** Academic Problems and Alcohol Expectancies for Low Risk and Hazardous Drinkers at T1 and T2

Academic Problems						
	T1 (*n* = 1995)			T2 (*n* = 1853)		
	Low risk	Hazardous	Total	Low risk	Hazardous	Total
	*N* (%)	*N* (%)	*N* (%)	*N* (%)	*N* (%)	*N* (%)
Been late for a class	87 (7.3)	242 (30.4)	329 (16.5)^a^	78 (6.7)	225 (32)	303 (16.3)^b^
Missed a class	129 (10.8)	310 (38.9)	439 (22)^a^	123 (10.6)	299 (42.6)	422 (22.7)^b^
Unable to concentrate in class	150 (12.5)	341 (42.8)	491 (24.6)^a^	146 (12.7)	320 (45.6)	466 (25.1)^b^
Failed to complete an assignment on time	22 (1.8)	96 (12)	118 (5.9)^a^	23 (1.9)	104 (14.8)	127 (6.8)^b^
Alcohol Expectancies						
	T1 (*n* = 2041)			T2 (*n* =		
	Low risk	Hazardous	Total	Low risk	Hazardous	Total
	*N* (%)	*N* (%)	*N* (%)	*N* (%)	*N* (%)	*N* (%)
Act more sociably	891 (72.4)	710 (86.4)	1592 (78)^a^	782 (66.8)	621 (86.6)	1403 (74.4)^b^
Find it easier to talk to people	880 (71.5)	680 (83.8)	1560 (76.4)^a^	806 (68.9)	626 (87.3)	1432 (75.9)^b^
Feel calmer/more relaxed	736 (59.8)	630 (77.7)	1366 (66.9)^a^	679 (58)	559 (78)	1238 (65.6)
Enjoy sex more	220 (17.9)	286 (35.3)	506 (24.8)^a^	207 (17.7)	261 (36.4)	468 (24.8)
Take risks	693 (56.3)	630 (77.7)	1323 (64.8)^a^	605 (51.7)	563 (78.5)	1168 (61.9)^b^
Be more aggressive	239 (19.4)	284 (35)	523 (25.6)^a^	210 (17.9)	276 (38.5)	486 (25.8)^b^
Feel more courageous	680 (55.3)	599 (73.9)	1279 (62.7)^a^	622 (53.2)	557 (77.7)	1179 (62.5)^b^
Act loud, boisterous, noisy	663 (53.9)	569 (70.2)	1232 (60.4)^a^	634 (54.2)	538 (75)	1172 (62.1)^b^
Have fun/good time	757 (61.5)	657 (81)	1414 (69.3)^a^	673 (57.5)	590 (82.3)	1263 (66.9)^b^

*T1* Significant between low risk and hazardous drinkers (*p* <0.001)^a^ *T2* Significant between low risk and hazardous drinkers (*p* <0.001)^b^

## Discussion

Although the intervention had minimal impact during the first year the cross sectional nature of this evaluation provided only a limited analysis and it was difficult to determine differential effects of specific strategies. It is likely the staggered approach to the implementation of the strategies may have also impacted the findings. In addition, population based behaviour change is a complex and slow process [34]. Across both time periods the proportion of young university students who reported consuming alcohol at hazardous levels was high. There was a slight but not significant decrease in the proportion of current drinkers who reported hazardous drinking at T1 (39.7 vs 38 %). Analyses of the sample over the two time periods found hazardous drinkers were significantly more likely to experience harms as a result of their own alcohol consumption, to experience second-hand harms and to witness harms as a result of other students drinking.

There were no significant differences between almost all demographics variables respondents at T2 were moderately significantly more likely to live with their parents. A New Zealand study found students living in university residential accommodation or shared house to drink at higher levels than those living with parents [3]. This study found most students to live with parents (T1 63.6 %; T2 60.3 %) to live with parents in comparison 18.9 % of respondents in the New Zealand study [3]. However these data are similar to that of another Australian university that found 54.9 % of respondent lived with parents [5]. The university in this study is largely a commuter university which impacts the proportion of young people living at home.

The intervention did not impact significantly on harms associated with alcohol consumption however the high levels of harms experienced by those classified as hazardous drinkers’ highlights the need for comprehensive strategies for this sub-group. While minimal intervention has been recognised as achieving some change, single strategy interventions are unlikely to have a significant impact on behaviour [34]. For example the web-based THRIVE alcohol brief intervention demonstrated positive short term results in reducing drinking frequency but found differences in alcohol-related harms to be non-significant [13]. Further exploration as how targeted promotion and personal referral could enhance this on line strategy for hazardous drinkers would be beneficial as well as research focusing on how such strategies can be extended to support long term behavioural change.

Alcohol expectancies remained similar across both time periods with students drinking at hazardous levels more likely to report positive expectancies. However, there was some reduction in the proportion of respondents reporting close friends consuming alcohol at T2. Associations between broad social motives, descriptive norms (the perception of what others do), personal drinking values and alcohol expectancies have been found to influence alcohol consumption and related harms among college students [43] and are consistent with Social Cognitive Theory which suggests behaviour is influenced by peers and expectations [14]. While there was little change between the two time periods this study found strong associations at both time periods between hazardous drinking and alcohol expectancies and associations between peer drinking and hazardous drinking, which demonstrate a need to focus on strategies that challenge descriptive and injunctive norms. Social acceptability of behaviours has been influenced positively through integrated and dedicated efforts at national, local and societal levels for issues such as smoking [34], however it is recognised that such changes take time and are most effective when a combination of educational, organisational, economic and political actions have been employed [35].

Many health promotion strategies are time consuming and complex and can be compromised if funded on short-term cycles [33]. This project had limited financial and personnel resources and as not all strategies of this intervention were implemented during the first year of the project. Organisational and policy actions have been identified as essential for effective health promotion [23, 34, 35] and the development and implementation of alcohol policy is one of the strategies yet to be implemented. Effective policies in communities and organisations need to be well developed with considerable emphasis on the adoption and implementation phase [44, 45]. Campus alcohol policy should reinforce and support responsible use of alcohol, reduce access, especially low cost and free alcohol, restrict heavy drinking on drinking premises and work to eliminate alcohol sponsorship on campus and in local communities to effect long term change [46]. From a population level it is recognised that comprehensive universal strategies that incorporate significant focus on policy are essential to effect change [47]. Policy implementation and promotion will be implemented as part of the ongoing intervention. In addition, strategies to build partnerships will be further developed in subsequent stages of this intervention. The establishment of partnerships and building capacity are time consuming and challenging however provide significant opportunities for change and will therefore be a focus of this intervention in the future [48].

The findings from the two data collection periods will help inform the development of additional strategies and provide evidence to support targeting specific sub-groups e.g. those drinking at hazardous levels. However, although the findings indicate that while more prevalent among hazardous drinkers, experienced, second-hand and witnessed harms and academic problems are of concern for both the low risk and hazardous drinkers. These data are similar to findings elsewhere [4, 5] and confirm the need for enhanced education and awareness of these issues.

This study has a number of limitations which should be considered when interpreting the findings. The cross sectional nature of the study precludes casual assumptions and rigorous intervention evaluation however when implementing and evaluating interventions in communities there are practical and financial constraints which may preclude more rigorous evaluation strategies [49, 50]. A cohort study, collecting data about recall of intervention strategies at T2, would provide a more rigorous evaluation, however was beyond the financial scope of this study [33]. Data were only collected from one university which may limit generalizability. Low response rates could have resulted in a non-respondent bias. Low response rates have been reported elsewhere [5]. It has been suggested non-respondents are more likely to participate in adverse health behaviours [3]. Self-report questionnaires may be subject to issues of social desirability, however comparisons with other studies suggest these data are reliable [4–6, 13].

## Conclusion

The findings over the two year period confirm the need for on-going alcohol interventions for young university students. After one year this study found relatively small changes in prevalence of hazardous drinking levels and maintenance of alcohol related harms, expectancies and behaviours, however, a number of the intervention strategies had not been implemented or had only been partially implemented. These findings do however provide the opportunity to refine and focus strategies. Harms and problems for students who report hazardous drinking are of concern and efforts should be made to ensure integrated and targeted strategies reach higher risk students and focus on specific issues such as drink driving and alcohol related unplanned sex. However there is also a need for universal strategies targeting all students and low risk drinkers as they too are exposed to alcohol harms within the drinking and social environment. Changing the culture of the university environment is a long term aim and to effect change a sustained combination of organisational actions, partnerships and educational actions is required.
